# Verifications

**Published:** 1889-05

**Authors:** A. H. Birdsall

**Affiliations:** Brooklyn, N. Y.


### VERIFICATIONS.

### Cases from Practice.

A. H. Birdsall, M. D., Brooklyn, N. Y.

Case I.—A young man came into my office complaining with what he termed a stabbing toothache. He said that he had taken cold the day previous in a tooth which was decayed, and since twelve o'clock that night had been suffering with severe *stabbing* or *jerking pains* coming at *intervals* of every *half-minute—single sharp stabs from the tooth up into* the *temple* and *ear*. This was the peculiarity of his toothache, and I do not think it exaggerated as regarded the frequency of these pains, as I noticed that he would as frequently jump and jerk back his head, significant of pain. I spent some ten minutes in looking for the simillimum in the young man's case. When I found in Repertory, under Baryta carb., the symptom, *single jerks in teeth* and and also in the provings " *Toothache* in *single jerks* in *decayed teeth* from cold, the pain reaching to temple and ear," I gave him a single dose of Baryta carb. (Fincke) dry on the tongue. At the expiration of *four minutes* the pains had entirely ceased, and the swelling and slight soreness of face and jaw gradually disappeared in the course of the next twenty-four hours.

CASE II.—Miss L. took a heavy cold in the head, which latterly developed into a severe neuralgia of the entire *left side* of head and face, involving eye of same side; the pains seemed to start from left occipital region and extend forward, pains sharp and tearing, especially in and around eye (ciliary neuralgia), causing much lachrymation, constant feeling of nausea, and occasional vomiting of bile, the least noise—even talking—aggravates the pain, and light is intolerable to the affected eye. She had been suffering in this manner for about three hours when I saw her. One dose of Spigelia$^{200}$ (B. & T.) stopped her pains in fifteen minutes.

CASE III.—Mr. R., aged twenty, while exercising in the gymnasium, accidentally received a severe bruise upon his right testicle. For a few minutes he suffered considerable pain, which gradually passed off, leaving a slight soreness for several days. Twelve days after the injury he sent for me in great haste, when I found him writhing in agonizing pain in his right (injured) testicle; the pain *sharp, cutting,* and running up spermatic cord to lower part of back and also *through scrotum* to *root* of *penis.* It was with difficulty that I obtained the latter and only characteristic symptom in his case, so great was his suffering. A dose of Conium$^{200}$ relieved his pain in five minutes, and at the end of twenty minutes it had entirely disappeared.

CASE IV.—Mrs. S., seven months enceinte, was taken one evening with intolerable neuralgic pains confined mostly to *left* side of head and face, apparently emanating from some carious tooth, although she was unable to locate it, as the teeth and gums of the entire left side were sore and sensitive, especially to *anything warm taken into* the *mouth.* I found her about eleven o'clock in the evening, walking rapidly up and down the room, moaning and crying, extremely *irritable,* decidedly *uncivil,* if not disposed to be quarrelsome. A few pellets of Chamomilla$^{cm}$

(Johnstone) gave relief in a very few minutes, and in less than half an hour I had the gratification of leaving her entirely free from suffering, and completely transformed into a state of supreme complacency of mind.

CASE V.—A gentleman came in my office, saying that he had been troubled for nine months with a diarrhœa that had baffled all the old-school doctors he had consulted, and that he would like to give Homœopathy a chance. Examination for his remedy seemed to elicit nothing really characteristic in his condition. Stools watery; yellowish at times, accompained with pain; again painless. Usually six or seven movements through the day, never at night; nothing particularly seemed to aggravate or bring on a movement. I was about to prescribe Nux-vom. and await development of symptoms, when he told me that he had noticed one thing throughout his trouble—that he invariably had a movement every morning *after* his *breakfast*, and also that if he ate no breakfast, he might have no movement for a couple of hours. One dose of *Thuja* and plenty of Sac-lac. cured this man in the course of five days, and he had no return of his trouble.

CASE VI.—Mr. L., aged forty-five, had been suffering with a severe, almost congestive type of chills and fever for two weeks, which he had been keeping partially under control with Quinine. His chill would usually occur every other day in the afternoon about four or five o'clock; the coldness, as he expressed it, seeming to penetrate every part of his body, causing him to shake terribly for nearly two hours, no amount of covering or external heat giving him any relief from the chill; his hands, feet, and face get very blue. Much restlessness and anxiety, and some thirst; drinking makes him feel more chilly. Fever follows, lasting most of the night, with *much thirst*. Chilliness during fever if moves or turns or raises covers; sweat with thirst follows heat, with desire to be uncovered. One dose of *Nux-vom.*$^{2000}$ immediately after a severe paroxysm completely intercepted the next chill, and cured without repetition of dose or paroxysm.

CASE VII.—A little girl of ten years of age had been troubled with an eczema situated on back of neck at border of hair for a period of two years; would get better and worse at times, but never entirely disappearing. There constantly exuded from it a secretion having the appearance of peach-gum. One dose of *Natr.-muriat.*$^{cm}$ (Swan) cured in two months without return—now one year since. Six years ago I cured a lady of hay fever (which had troubled her every summer for a number of years) with several doses of the same remedy, solely on the strength of the above indications.

CASE VIII.—A theological student had been troubled with dyspepsia and chronic constipation for several years, at times giving him so much trouble that he would have to relinquish his studies. Appetite generally good, but slight errors in diet were apt to bring on an attack of gastralgia. Much tenderness, and at times bloating in the epigastrium, so that the clothing would be uncomfortable and painful; frequent spells of empty, weak feelings in pit of stomach. Food always lay heavy in stomach, producing sensation like a stone lying there, attended with good deal of belching. Generally awakens in the morning with dull feeling in head and pain across the forehead, bowels inactive—always had to take cathartics to get them to move. If not would use enemata, when the pain and straining would become almost intolerable, the stools being large, dry, and, as he expressed it, hard as though baked. He also complained at times of pain and soreness of right side, felt mostly in stepping heavily in walking, and deep breathing, coughing, or sneezing. Three doses of Bryonia 2000 (Jen.) at intervals of ten days cured permanently in three months.

